# Supplementary material for: Pricing decision and channel selection of fresh agricultural products dual-channel supply chain based on blockchain
Source: PLoS One. 2024 Mar 28;19(3):e0297484. doi: 10.1371/journal.pone.0297484 (PMC10977692; doi:10.1371/journal.pone.0297484)
Supplement: S1 Appendix — (DOCX) [file pone.0297484.s001.docx]

**S1 Appendix**

**Proof of 4.1** Equilibrium solution of NS model Solving by inverse order induction, substituting equations (5) and (6) into equation (7) to find the first order partial derivative of $p_{r}^{NS}$, we have $\frac{\partial\pi_{R}^{NS}}{\partial p_{r}^{NS}}=\theta(t_{0})(sa-2p_{r}^{NS}+\beta p_{e}^{NS}+\omega)$. Then we have $\frac{\partial^{2}\pi_{R}^{NS}}{\partial p_{r}^{NS2}}=-2\theta(t_{0})<0$, so the retailers' profits $\pi_{R}^{NS}$ is a concave function on the traditional channel sales price $p_{r}^{NS}$. Thus, the response function of $p_{r}^{NS}(p_{e}^{NS},\omega_{r}^{NS})$ to the wholesale price $\omega_{r}^{NS}$ and the online channel price $p_{e}^{NS}$ can be found form $\frac{\partial\pi_{R}^{NS}}{\partial p_{r}^{NS}}=0$, and substituting the response function into equation (6), we can obtain the manufacturer's profit $\pi_{M}^{NS}$ as a function of $\omega_{r}^{NS}$ and $p_{e}^{NS}$. So we can obtain its Hesse matrix as ： $H_{1}=\left[\begin{matrix} \frac{\partial^{2}\pi_{M}^{NS}}{\partial p_{e}^{NS2}} & \frac{\partial^{2}\pi_{M}^{NS}}{\partial p_{e}^{NS}\partial\omega_{r}^{NS}} \\ \frac{\partial^{2}\pi_{M}^{NS}}{\partial\omega_{r}^{NS}\partial p_{e}^{NS}} & \frac{\partial^{2}\pi_{M}^{NS}}{\partial\omega_{r}^{NS2}} \end{matrix}\right]=\left[\begin{matrix} -\theta(t_{0})(2-\beta^{2}) & \beta\theta(t_{0}) \\ \beta\theta(t_{0}) & -\theta(t_{0}) \end{matrix}\right]$. Thus, we have $\left|H_{1}\right|=2\theta(t_{0})^{2}(1-\beta^{2})>0$, where $\theta(t_{0})\in[0,1],0<\beta<1$, and $\frac{\partial^{2}\pi_{M}^{NS}}{\partial p_{e}^{NS2}}<0$. Then, its Hessian matrix is negative definite, the manufacturer's profit $\pi_{M}^{NS}$ is a joint concave function for $\omega_{r}^{NS}$ and $p_{e}^{NS}$. Associating equation $\frac{\partial\pi_{M}^{NS}}{\partial p_{e}^{NS}}=\frac{\partial\pi_{M}^{NS}}{\partial\omega_{r}^{NS}}=0$, we can obtain the optimal solutions $\omega_{r}^{NS*}$ and $p_{e}^{NS*}$, and substituting them into the reaction function $p_{r}^{NS}(p_{e}^{NS},\omega_{r}^{NS})$ to get $p_{r}^{NS*}$.

Therefore, in the dual-channel model (NS) of direct vendor online sales without applying blockchain, the inverse order solution method is used to obtain the optimal online channel sales price, wholesale price and traditional channel sales price:

|  | (A1) |
| --- | --- |
|  | (A2) |
| $p_{r}^{NS*}=\frac{(3-\beta^{2})sa+2(1-s)\beta a}{4(1-\beta^{2})}+\frac{(1+\beta)A}{2}+\frac{\beta c_{z}}{4}$ | (A3) |

At this point, the optimal demand for traditional and online channels yields the following results:

| $D_{r}^{NS*}=\frac{\theta(t_{0})}{4}(sa+\beta c_{z}-2A(1-\beta))$ | (A4) |
| --- | --- |
| $D_{e}^{NS*}=\frac{\theta(t_{0})}{4}(s\beta a+2(1-s)a-(2-\beta^{2})c_{z}-2A(2-\beta-\beta^{2}))$ | (A5) |

The profits of traditional retailers and manufacturers are respectively:

| $\pi_{R}^{NS*}=\frac{\theta(t_{0})}{16}(sa+\beta c_{z}-2A(1-\beta))^{2}$ | (A6) |
| --- | --- |
| $\begin{matrix} \pi_{M}^{NS*}=\frac{\theta(t_{0})}{8(1-\beta^{2})}(s^{2}(1+\beta^{2})a^{2}+4(1-s)s\beta a^{2}+2(1-s)^{2}a^{2})+\frac{\theta(t_{0})}{8}((2-\beta^{2})(2A+c_{z})^{2} \\ +4A^{2}-4saA-2\beta(sa+2A)(2A+c_{z})-4(1-s)a(2A+c_{z})) \end{matrix}$ | (A7) |

The total profit of the dual-channel supply chain system is:

| $\begin{matrix} \pi_{C}^{NS*}=\frac{\theta(t_{0})}{16(1-\beta^{2})}(8\beta s(1-s)a^{2}+4\beta(1-s)^{2}a^{2}+(3+\beta^{2})s^{2}a^{2})+\frac{\theta(t_{0})}{16}((4-\beta^{2}) \\ (2A+c_{z})^{2}+12A^{2}-4(4-s)aA-2\beta(sa+6A)(2A+c_{z})-8(1-s)a(2A+c_{z})) \end{matrix}$ | (A8) |
| --- | --- |

Where , .

**Proof of 4.2, 5.1, 5.2** The equilibrium solution solving process for the ND, BS and BD models is the same as that for the 4.1 NS model and is therefore omitted.

**Poof of Proposition 1** Find the first order derivative of $\omega_{r}^{NS*}$ with respect to yields , where , , by the same token, we can prove the other.

**Poof of Proposition 2** (a) The first-order derivative of $p_{e}^{NS*}$, $p_{r}^{NS*}$, $\omega_{r}^{NS*}$ with respect to $s$ can be obtained separately as follows $\frac{\partial p_{e}^{NS*}}{\partial s}=-\frac{a}{2(1+\beta)}<0$, $\frac{\partial p_{r}^{NS*}}{\partial s}=\frac{(3+\beta)a}{4(1+\beta)}>0$, $\frac{\partial\omega_{r}^{NS*}}{\partial s}=\frac{a}{2(1+\beta)}>0$. Similarly $\frac{\partial p_{e}^{ND*}}{\partial s}<0$, $\frac{\partial p_{r}^{ND*}}{\partial s}>0$, $\frac{\partial\omega_{e}^{ND*}}{\partial s}<0$, $\frac{\partial\omega_{r}^{ND*}}{\partial s}>0$. (b) From the results above, we can obtain $\omega_{e}^{ND*}-\omega_{r}^{ND*}=\frac{(1-2s)a}{2(1+\beta)}$, it follows that when $0<s\leq1/2$, there is $\frac{(1-2s)a}{2(1+\beta)}>0$, hence $\omega_{e}^{ND*}\geq\omega_{r}^{ND*}$; when $1/2<s<1$, there is $\frac{(1-2s)a}{2(1+\beta)}<0$, hence $\omega_{e}^{ND*}<\omega_{r}^{ND*}$. (c) The first-order derivative of $p_{e}^{NS*}$, $p_{r}^{NS*}$, $\omega_{r}^{NS*}$ with respect to $c_{z}$ can be obtained , , $\frac{\partial\omega_{r}^{NS*}}{\partial c_{z}}=0$.

**Poof of Proposition 3** (a) The first-order derivative of $\pi_{R}^{NZ*}$ with respect to $s$ can be obtained $\frac{\partial\pi_{R}^{NS*}}{\partial s}=\frac{\theta(t_{0})a}{8}(sa\text{+}\beta c_{z}-2A(1-\beta))>0$, the same can be obtained $\frac{\partial\pi_{R}^{ND*}}{\partial s}>0$and $\frac{\partial\pi_{E}^{ND*}}{\partial s}<0$. (b) The same can be obtained $0<s\leq1/2$, $\frac{\partial\pi_{M}^{ND*}}{\partial s}\leq0$, the opposite $\frac{\partial\pi_{M}^{ND*}}{\partial s}>0$ . (c) The first-order derivative of $\pi_{M}^{NS*}$ with respect to $s$ can be obtained . So when , there is $\frac{\partial\pi_{M}^{NS*}}{\partial s}\leq0$, the opposite $\frac{\partial\pi_{M}^{NS*}}{\partial s}>0$.

**Poof of Proposition 4** From the results above, we can obtain $p_{e}^{NS*}-p_{e}^{ND*}=-\frac{2(1-s)a+s\beta a}{2(4-\beta^{2})}+\frac{A(1-\beta)}{2-\beta}+\frac{c_{z}}{2}$ , similarly, we can obtain $p_{r}^{NS*}-p_{r}^{ND*}$ and $\pi_{R}^{NS*}-\pi_{R}^{ND*}$. So it can be known when $0<c_{z}\leq\frac{2(1-s)a+s\beta a}{2(4-\beta^{2})}-\frac{A(1-\beta)}{2-\beta}$, there are $p_{e}^{NS*}\leq p_{e}^{ND*}$, $p_{r}^{NS*}\leq p_{r}^{ND*}$, $\pi_{R}^{NS*}\leq\pi_{R}^{ND*}$; and when $c_{z}>\frac{2(1-s)a+s\beta a}{4-\beta^{2}}-\frac{A(1-\beta)}{2-\beta}$, there are $p_{e}^{NS*}>p_{e}^{ND*}$, $p_{r}^{NS*}>p_{r}^{ND*}$, $\pi_{R}^{NS*}>\pi_{R}^{ND*}$. Similarly, when $c_{z}>\frac{(4-\beta^{2}+\sqrt{8-2\beta^{2}})(s\beta a+2(1-s)a)}{(2-\beta^{2})(4-\beta^{2})}-\frac{2A(2-\beta-\beta^{2})}{(2-\beta^{2})(4-\beta^{2})}$, there is $\pi_{M}^{NS*}<\pi_{M}^{ND*}$, and vice versa $\pi_{M}^{NS*}\geq\pi_{M}^{ND*}$.

**Poof of Proposition 5** The first-order derivative of $\theta(\tau)$, , $c_{\tau}$ with respect to $\tau$ can be obtained $\frac{\partial\theta(\tau)}{\partial\tau}=\theta(0)\frac{2(t_{0}(1-\tau))}{T^{2}}=\mu$, , $\frac{\partial c_{\tau}}{\partial\tau}=k_{\tau}\tau$. Finding the second order derivative of $p_{e}^{BS*}$ with respect to $\tau$ yields . Similarly, $\frac{\partial^{2}p_{r}^{J*}}{\partial\tau^{2}}>0$, $\frac{\partial^{2}p_{e}^{BD*}}{\partial\tau^{2}}>0$, $\frac{\partial^{2}w_{r}^{J*}}{\partial\tau^{2}}>0$, $\frac{\partial^{2}\omega_{e}^{BD*}}{\partial\tau^{2}}>0$; $\frac{\partial^{2}D_{e}^{J*}}{\partial\tau^{2}}<0$, $\frac{\partial^{2}D_{r}^{J*}}{\partial\tau^{2}}<0$; $\frac{\partial^{2}\pi_{R}^{J*}}{\partial\tau^{2}}<0$, $\frac{\partial^{2}\pi_{M}^{J*}}{\partial\tau^{2}}<0$, $\frac{\partial^{2}\pi_{E}^{BD*}}{\partial\tau^{2}}<0$.

**Poof of Proposition 6** (a) The first-order derivative of $p_{e}^{BS*}$, $p_{r}^{BS*}$, $\omega_{r}^{BS*}$, $p_{e}^{BD*}$, $p_{r}^{BD*}$, $\omega_{e}^{BD*}$ and $\omega_{r}^{BD*}$ with respect to $s$ can be obtained $\frac{\partial p_{e}^{BS*}}{\partial s}=-\frac{a}{2(1+\beta)}<0$, $\frac{\partial p_{r}^{BS*}}{\partial s}=\frac{(3+\beta)a}{4(1+\beta)}>0$, $\frac{\partial\omega_{r}^{BS*}}{\partial s}=\frac{a}{2(1+\beta)}>0$, $\frac{\partial p_{e}^{BD*}}{\partial s}=-\frac{a}{1+\beta}<0$, $\frac{\partial p_{r}^{BD*}}{\partial s}=\frac{a}{1+\beta}>0$, $\frac{\partial\omega_{e}^{BD*}}{\partial s}=-\frac{a}{2(1+\beta)}<0$, $\frac{\partial\omega_{r}^{BD*}}{\partial s}=\frac{a}{2(1+\beta)}>0$. (b) From the results above, we can obtain$p_{e}^{BS*}-\omega_{r}^{BS*}=\omega_{e}^{BD*}-\omega_{r}^{BD*}=\frac{(1-2s)a}{2(1+\beta)}$, it follows that when $0<s\leq1/2$, there is $\frac{(1-2s)a}{2(1+\beta)}>0$, therefore $p_{e}^{BS*}\geq w_{r}^{BS*}$, $\omega_{e}^{BD*}\geq\omega_{r}^{BD*}$.

**Poof of Proposition 7** From the results above, we can obtain $p_{e}^{BS*}-p_{e}^{BD*}=-\frac{2(1-s)a+s\beta a+(2+\beta)\delta\tau}{2(4-\beta^{2})}+\frac{B(1-\beta)}{2-\beta}$, the same can be done at $p_{r}^{BS*}-p_{r}^{BD*}$, $D_{r}^{BS*}-D_{r}^{BD*}$, $D_{e}^{BS*}-D_{e}^{BD*}$, $\pi_{R}^{BS*}-\pi_{R}^{BD*}$, $\pi_{M}^{NS*}-\pi_{M}^{ND*}$, so that $p_{e}^{BD*}-p_{e}^{BS*}=0$, $p_{r}^{BS*}-p_{r}^{BD*}=0$, $D_{r}^{BS*}-D_{r}^{BD*}=0$, $D_{e}^{BS*}-D_{e}^{BD*}=0$, $\pi_{R}^{BS*}-\pi_{R}^{BD*}=0$, $\pi_{M}^{NS*}-\pi_{M}^{ND*}=0$ can be obtained from , and since the constant holds, we have $p_{e}^{BS*}<p_{e}^{BD*}$, $p_{r}^{BS*}<p_{r}^{BD*}$, $D_{r}^{BS*}<D_{r}^{BD*}$, $\pi_{R}^{BS*}<\pi_{R}^{BD*}$, $D_{e}^{BS*}>D_{e}^{BD*}$, $\pi_{M}^{BS*}>\pi_{M}^{BD*}$.

**Poof of Proposition 8** (a) Let $\omega_{r}^{BS*}-\omega_{r}^{NS*}=\frac{\delta\tau}{2(1-\beta)}+\frac{1}{2}(c_{\tau}-C)$, so when $c_{\tau}>C-\frac{\delta\tau}{1-\beta}$, there is $\omega_{r}^{BS*}>\omega_{r}^{NS*}$; and when $0<c_{\tau}\leq C-\frac{\delta\tau}{1-\beta}$, there is $\omega_{r}^{BS*}<\omega_{r}^{NS*}$. (b) Let $p_{r}^{BS*}-p_{r}^{NS*}=\frac{1+\beta}{4}(c_{\tau}-C)-\frac{\beta c_{z}}{4}$, so when $c_{\tau}>C+\frac{\beta c_{z}}{1+\beta}-\frac{(3-\beta)\delta\tau}{1-\beta}$, there is $p_{r}^{BS*}>p_{r}^{NS*}$; and when $0<c_{\tau}\leq C+\frac{\beta c_{z}}{1+\beta}-\frac{(3-\beta)\delta\tau}{1-\beta}$, there is $p_{r}^{BS*}\leq p_{r}^{NS*}$. (c) Similarly, we can prove that when $c_{\tau}>C-\frac{\delta\tau}{1-\beta}+c_{z}$, there is $p_{e}^{BS*}>p_{e}^{NS*}$; and when $0<c_{\tau}\leq C-\frac{\delta\tau}{1-\beta}+c_{z}$, there is $p_{e}^{BS*}\leq p_{e}^{NS*}$.

**Poof of Proposition 9** (a) If you do $\pi_{R}^{BS*}\geq\pi_{R}^{NS*}$, you need to satisfy , so you can get when , there is $\pi_{R}^{BS*}\geq\pi_{R}^{NS*}$; and vice versa for $\pi_{R}^{BS*}<\pi_{R}^{NS*}$; (b) The same can be found when $0<c_{\tau}\leq c_{\tau}^{M*}$, $\pi_{M}^{BS*}\geq\pi_{M}^{NS*}$, and vice versa for $\pi_{M}^{BS*}<\pi_{M}^{NS*}$.

**Poof of Proposition 10** (a) From the results above, we can obtain $\omega_{r}^{BD*}-\omega_{r}^{ND*}=\omega_{e}^{BD*}-\omega_{e}^{ND*}=\frac{\delta\tau}{2(1-\beta)}-\frac{1}{2}C+\frac{c_{\tau}}{2}$, so there is when $c_{\tau}>C-\frac{\delta\tau}{1-\beta}$, there are $\omega_{r}^{BD*}>\omega_{r}^{ND*}$, $\omega_{e}^{BD*}>\omega_{e}^{ND*}$;and when $0<c_{\tau}\leq C-\frac{\delta\tau}{1-\beta}$, there is $\omega_{r}^{BD*}\leq\omega_{r}^{ND*}$, $\omega_{e}^{BD*}\leq\omega_{e}^{ND*}$. (b) The same we can obtain $p_{r}^{BD*}-p_{r}^{ND*}=p_{e}^{BD*}-p_{e}^{ND*}=c_{\tau}-C+\frac{(6-\beta-2\beta^{2})\delta\tau}{(1-\beta)(2+\beta)}$ when $c_{\tau}>C-\frac{(6-\beta-2\beta^{2})\delta\tau}{(1-\beta)(2+\beta)}$, there are $p_{r}^{BD*}>p_{r}^{ND*}$, $p_{e}^{BD*}>p_{e}^{ND*}$; and when $0<c_{\tau}\leq C-\frac{(6-\beta-2\beta^{2})\delta\tau}{(1-\beta)(2+\beta)}$, there is $p_{r}^{BD*}\leq p_{r}^{ND*}$, $p_{e}^{BD*}\leq p_{e}^{ND*}$.

**Poof of Proposition 11** From the results above, we can obtain $\pi_{R}^{BD*}-\pi_{R}^{ND*}=\theta(t_{1})(\frac{2(1-s)a+s\beta a+(2+\beta)\delta\tau}{2(4-\beta^{2})}-\frac{B(1-\beta)}{2-\beta})^{2}-\theta(t_{0})(\frac{2(1-s)a+s\beta a}{2(4-\beta^{2})}-\frac{A(1-\beta)}{2-\beta})^{2}$, so when , there is $\pi_{R}^{BD*}\geq\pi_{R}^{ND*}$; conversely, there is $\pi_{R}^{BD*}<\pi_{R}^{ND*}$, and the same can be proved when $0<c_{\tau}\leq c_{\tau}^{ED*}$, $\pi_{E}^{BD*}\geq\pi_{E}^{ND*}$; when $c_{\tau}>c_{\tau}^{ED*}$, $\pi_{E}^{BD*}<\pi_{E}^{ND*}$and when $0<c_{\tau}\leq c_{\tau}^{M*}$, $\pi_{M}^{BD*}\geq\pi_{M}^{ND*}$; when $c_{\tau}>c_{\tau}^{M*}$, $\pi_{M}^{BD*}<\pi_{M}^{ND*}$.
