# Supplementary material for: Pricing decision and channel selection of fresh agricultural products dual-channel supply chain based on blockchain
Source: PLoS One. 2024 Mar 28;19(3):e0297484. doi: 10.1371/journal.pone.0297484 (PMC10977692; doi:10.1371/journal.pone.0297484)
Supplement: Attachment — Submitted filename: Response to Reviewers.docx [file pone.0297484.s002.docx]

Response to Academic editor

Dear academic editor,

Thank you very much for your kind review and suggestions. According to your suggestions, we have made careful modification of our manuscript.

Thank you again for your positive comments and valuable suggestions to improve the quality of our manuscript.

With kind regards.

Di Wang

**Journal requirements:**

**Point 1:** Thank you for stating the following financial disclosure:

“Funding: Henan Province Philosophy and Social Science Planning Project（http://www.hnpopss.gov.cn/）Zhifang Li & Di Wang(2022CZH016); Henan Polytechnic University Basic Research Business Fund Special Project (http://www.hpu.edu.cn) Di Wang (SKJYB2023-13); Henan Polytechnic University Young Backbone Teacher Funding Scheme (http://www.hpu.edu.cn) Di Wang (2022XQG-14); The funders had no role in study design, data collection and analysis, decision to publish, or preparation of the manuscript.”

At this time, please address the following queries:

a) Please clarify the sources of funding (financial or material support) for your study. List the grants or organizations that supported your study, including funding received from your institution.

b) State what role the funders took in the study. If the funders had no role in your study, please state: “The funders had no role in study design, data collection and analysis, decision to publish, or preparation of the manuscript.”

c) If any authors received a salary from any of your funders, please state which authors and which funders.

d) If you did not receive any funding for this study, please state: “The authors received no specific funding for this work.”

Please include your amended statements within your cover letter; we will change the online submission form on your behalf.

**Response 1:** Thank you for your comment. We have added funding information to the latest cover letter. The detailed information is as follows:

(1) Funding Institution: Henan Provincial Department of Philosophy and Social Sciences Planning; Fund Project: Henan Provincial Department of Philosophy and Social Sciences Planning Project (2022CZH016) (http://www.hnpopss.gov.cn/); Project Leader: Di Wang.

(2) Funding Institution: Henan Polytechnic University; Fund Project: Henan Polytechnic University Basic Research Business Special Project (SKJYB2023-13) (http://www.hpu.edu.cn); Project Leader: Di Wang.

(3) Funding Institution: Henan Polytechnic University; Fund Project: Henan Polytechnic University Young Backbone Teacher Support Program (2022XQG-14) (http://www.hpu.edu.cn); Project Leader: Di Wang

The funder, Wang Di, as the first author and corresponding author of this article, took on data analysis, preparation of the manuscript, and publication decision-making responsibilities in this study.

**Point 2:** Please include captions for your Supporting Information files at the end of your manuscript, and update any in-text citations to match accordingly.

**Response 2:** Thank you for your comment. We have revised captions for the Supporting Information files at the end of the manuscript, and updated all the in-text citations to match accordingly the manuscript as required in accordance with the journal’s guidelines.

**Additional Editor Comments:**

**Point 1:** Firstly, I would like to highlight that the current format of your manuscript does not adhere to our journal's guidelines for authors. Before proceeding with the revision, I kindly request that you review the guidelines and make necessary adjustments to ensure compliance.

**Response 1:** Thank you for your comment. We have revised the previous manuscript as required in accordance with the journal’s guidelines.

**Point 2:** Next, it is essential to revise the introduction section to more effectively elucidate the primary motivations, needs, and benefits of your research. This will provide readers with a clearer understanding of the significance of your paper.

**Response 2:** Thank you for your positive comments and valuable suggestions to improve the quality of our manuscript. In view of the main motivation, needs and significance of this study, we have revised the introduction section of this paper.

**Point 3:** The literature review in your manuscript overlooks several relevant articles in the field, which I recommend incorporating:

Asghari, M., Afshari, H., Mirzapour Al-e-hashem, S. M. J., Fathollahi-Fard, A. M., & Dulebenets, M. A. (2022). Pricing and advertising decisions in a direct-sales closed-loop supply chain. Computers & Industrial Engineering, 171, 108439.

Edalatpour, M. A., Mirzapour Al-e-Hashem, S. M. J., & Fathollahi-Fard, A. M. (2023). Combination of pricing and inventory policies for deteriorating products with sustainability considerations. Environment, Development and Sustainability, 1-41.

Fathollahi-Fard, A. M., Dulebenets, M. A., Hajiaghaei–Keshteli, M., Tavakkoli-Moghaddam, R., Safaeian, M., & Mirzahosseinian, H. (2021). Two hybrid meta-heuristic algorithms for a dual-channel closed-loop supply chain network design problem in the tire industry under uncertainty. Advanced engineering informatics, 50, 101418.

**Response 3:** Thank you for your comment and valuable suggestions. As suggested by the reviewer, we have added the relevant references to the manuscript. E.g.: Reference [16], [17] and [23].

**Point 4:** To clarify and justify the novelty of your work in comparison with these published works, I recommend providing a comparative table.

**Response 4:** Thank you for your comment and valuable suggestions. Based on your recommendations, we have compared this paper with the cited published articles, and the results are presented in Table 1 of the revised version.

**Point 5:** In Section 3, before subsection 3.1, please provide justifications and clarifications regarding the objectives of this section and the rationale for its division into different subsections. Additionally, establish links between these subsections. The same applies to Section 4 and Section 5.

**Response 5:** Thank you for your comment. We have added transitional paragraphs between the various sections of the article, elucidating the research objectives, content, and focal points of each section, serving as bridges between them.

**Point 6:** Section 4 holds significant importance in your paper. However, it requires better presentation. Many formulations lack sufficient explanation, which may hinder readers' understanding. I suggest providing more detailed explanations to improve clarity.

**Response 6:** Thanks for your comments and valuable suggestions. According to your suggestions, we have supplemented and modified the Section 4 of this article. For the formula solving part, we add a detailed solution process. We also give a more detailed explanation of the explanatory part of the proposition. Based on your feedback, we have made enhancements and revisions to Section 4 of this article. In the formula-solving segment, we have included a comprehensive step-by-step solution process. Additionally, we have provided a more in-depth explanation of the propositions.

**Point 7:** Furthermore, it is essential for the authors to include a table comparing the models and incorporate charts to analyze the behavior of their models in a comparative study.

**Response 7:** Thank you for your comment. In accordance with your recommendations, we have conducted a model comparison in Tables 3 and 4 within this article, presenting the optimal decisions for each model. Furthermore, we have incorporated Figures 2-4 into the numerical analysis section of the paper to investigate the effects of various parameters on the profits of supply chain participants across different models. Specifically, Figure 2-4 respectively delve into blockchain unit variable cost, the level of blockchain technology investment, and consumer channel preference on the profits of supply chain members.

**Point 8:** Finally, in the conclusion section, it is crucial to discuss the limitations of your research and propose potential areas for future research.

**Response 8:** Thank you for your comment. We have revised the conclusion section of this paper to discuss the limitations of this research and propose potential areas for future research.

Response to Reviewer 1 Comments

Dear reviewer,

Thank you very much for your kind review and suggestions. According to your suggestions, we have made careful modification of our manuscript.

Thank you again for your positive comments and valuable suggestions to improve the quality of our manuscript.

With kind regards.

Di Wang

**Point 1:** The motivation for the study is not clearly articulated. It would be helpful to provide a more compelling rationale for why this research is necessary and how it contributes to the existing literature. Additionally, the lack of innovation in the paper is evident as mainly focuses on applying blockchain technology to the existing supply chain without introducing any novel concepts or approaches.

**Response 1:** Thank you for your comments on our article. At your suggestion, we have made supplementary revisions to the abstract, introduction, and conclusion sections to further elucidate the novelty and research significance of this article.

The necessity and significance of this paper are primarily reflected in the following aspects: Most of the literature on the dual-channel supply chain of fresh agricultural products focuses on the impact of dual losses on supply chain decisions, while there is limited research on the pricing and channel selection in the context of fresh agricultural product supply chains based on blockchain technology. There is even less literature exploring the influence of blockchain technology on pricing and channel selection in dual-channel agricultural product supply chains. In the literature on the impact of blockchain technology on supply chain decisions, the discussion is limited to its effects on traditional single-channel supply chain decisions. Therefore, this paper selects two common dual-channel supply chain structures, online direct sales and online distribution, and combines specific parameters such as manufacturer's direct selling costs and consumer channel preferences. It further introduces blockchain-specific parameters such as the level of blockchain technology investment and blockchain variable costs. Four dual-channel agricultural product supply chain models are constructed, including two without blockchain technology and two with blockchain technology. These models are developed to analyze the optimal pricing and channel selection strategies under each mode using manufacturer-led Stackelberg game analysis. Finally, the rationality of the theoretical model is validated through numerical simulations. The aim is to provide theoretical guidance for the practical application of blockchain technology in the dual-channel supply chain of fresh agricultural products.

**Point 2:** The discussion section is quite brief and lacks depth. It would be beneficial to expand on the findings and provide a more comprehensive analysis of the results. Furthermore, the paper would benefit from a critical evaluation of the limitations and implications of the study.

**Response 2:** Thank you for your comments on our article. Based on your suggestions, in Section 6, we have provided a more comprehensive supplementation and exploration of the proposed propositions, and in Section 7, we have conducted a more detailed numerical simulation to evaluate and validate the impact of parameters such as the level of blockchain technology investment, blockchain variable costs, consumer channel preferences, etc., on supply chain decision-making. Simultaneously, we have expanded Section 8 of the article to summarize additional key findings and present relevant decision recommendations for different stakeholders. Furthermore, we have provided a forward-looking perspective on the limitations of this study.

**Point 3:** The language and writing style of the paper need improvement. There are grammatical errors and awkward sentence structures throughout the manuscript. I recommend having the paper proofread by a native English speaker to enhance its readability and clarity.

**Response 3:** Thank you for your comment and valuable suggestions. Based on your suggestions, we have proofread the entire article, corrected grammar errors, and adjusted sentence structures.

We have also responded to your questions below.

**Question 1:** How does the proposed dual-channel structure differ from traditional supply chain models? What are the advantages and disadvantages of each model?

**Response 1:** Thank you for your question. In this study, we introduce blockchain technology into a dual-channel agricultural supply chain dominated by manufacturers, considering parameters such as the level of blockchain technology investment, blockchain variable costs, and consumer channel preferences. We establish a Stackelberg game model to explore the impact of adopting blockchain and not adopting blockchain on pricing and channel decisions within the two dual-channel structures. We also conduct a comparative analysis between online direct sales and online distribution models.

For the NS, ND, BS and BD models proposed in this paper, their advantages and disadvantages can be summarized as follows:

NS model: Under a certain online direct selling cost, manufacturers can through online channels to expand demand at a lower price, increase profits. However, this intensifies the channel competition with traditional retailers, which will lead to the loss of profits of traditional retailers.

ND model: Manufacturers using online distribution, relatively online direct sales model can reduce the competition between the two channels. However, this is not an optimal choice for the manufacturer himself.

BS model: The adoption of blockchain can improve and transform the existing enterprise network platform, thus saving the online direct selling cost of manufacturers. But in its place are the costs of blockchain technology. In addition, when the cost of blockchain technology is within a certain threshold, the profits of supply chain members and the overall can be effectively improved.

BD model: The adoption of blockchain technology will lead to an increase in the cost of manufacturers, but when the cost is within a certain threshold, compared with the ND model, the profits of supply chain members and the overall can be effectively improved under this model.

**Question 2:** Can you elaborate on the methodology used to construct the four dual-channel supply chain decision models? How were the optimal strategies for pricing and channel selection determined?

**Response 2:** Thank you for your question. In this article, we mainly use the method of game theory, guided by Stackelberg's game theory. Of these four models, a two-stage game is constructed, involving manufacturers and traditional and online retailers.

In the block before and after the chain technology builds the use of online direct marketing NS and BS model, the decision as follows: the first stage, the manufacturers in order to maximize their own interests as the goal, decision-making and traditional channels of the retail price of the online channel wholesale price. In the second stage, the traditional retailer decides the retail price in the traditional channel.

In the block before and after the chain technology builds the use of online distribution ND and BD model, the decision as follows: the first stage, the manufacturers in order to maximize their own interests as the goal, decides the wholesale price of online channel and traditional channel. In the second stage, the traditional and Internet retailers simultaneously decide the retail prices in their respective channels.

After the game model is constructed, the optimal pricing and profit under each model are obtained by backward induction. The best strategy for pricing and channel selection is then determined by parametric analysis and comparison between models. For example, by comparing NS, ND, BS and BD models respectively in 4.3 and 5.3 of this paper, the cost critical values of pricing and channel selection of manufacturers and retailers under the two models can be obtained. It is then possible to determine which channel model manufacturers should choose when the relevant costs are within the threshold and how manufacturers and retailers should price.

**Question 3:** The paper mentions that the introduction of blockchain technology can improve consumer trust and shorten circulation time. Can you provide more details on how blockchain achieves these outcomes in the context of the fresh agricultural products supply chain?

**Response 3:** Thank you for your question. Our answer to your question is as follows: The characteristics of blockchain technology, such as decentralization, data immutability, and security transparency, can meet consumers' requirements for the safety and freshness of fresh agricultural products. This encourages retailers to not only seize the consumer market by opening up dual-channel models but also consider how to incorporate blockchain technology to achieve profit optimization. Additionally, the "smart contract" technology of blockchain can reduce transaction times, thereby decreasing double losses, while simultaneously ensuring the safety and transparency of fresh agricultural products, ultimately enhancing consumer trust.

**Question 4:** The paper states that the selling price rises with direct sales costs or blockchain change costs in both dual-channel structure models. Can you explain the underlying reasons for this relationship? How do these costs impact the profitability of the supply chain members?

**Response 4:** Thank you for your comment. The underlying reasons for this relationship is that as the cost of direct sales or blockchain increases, manufacturers and retailers need to transfer part of the cost to ensure their own interests, so they need to transfer the cost to consumers by increasing the selling price.

When blockchain technology is not adopted, when manufacturers open up online direct sales channels for online sales, they have certain direct sales advantages in the online direct sales model compared with the network distribution model, and can conduct online sales at a lower price. At this time, when the cost of direct sales is small, the manufacturer's network channel can form a situation of small profits and high sales, thereby increasing its profitability. At the same time, due to the lower cost of direct sales, manufacturers will not pass on costs to traditional retailers by raising wholesale prices, but because the low-price sales behavior of manufacturers' network channels will shift some consumers from offline to online, it will make traditional retailers less profitable.

Similarly, when the variable cost of blockchain is high, fresh agricultural products manufacturers will transfer part of the cost to downstream retailers and consumers by increasing wholesale prices and electronic sales prices, and retailers will also transfer part of the costs to consumers again by increasing the dual-channel selling price. In the end, the high premium leads to a decrease in the demand of the dual channels, and the positive effect of the increase in net benefit per unit is less than the negative effect caused by the decrease in demand, thus reducing the profit capacity of both parties.

**Question 5:** The paper mentions that the ability of each member and system to bear the variable cost of blockchain technology varies. Could you elaborate on the factors that influence this ability and how it affects pricing and profitability?

**Response 5:** Thank you for your comment. In this paper, we first assume that the blockchain technology variable cost is solely borne by the manufacturer. In the process of the game, the manufacturer transfers the variable cost of blockchain technology to the traditional retailer by increasing the wholesale price, and the traditional retailer can transfer the cost to the consumer by increasing the retail price. Therefore, when the variable cost of blockchain technology is within a certain range, the profit of supply chain members can be effectively improved. However, different dual-channel structures and consumer channel preferences will change the ability of each member and system to bear the variable costs of blockchain technology.

Different dual-channel structures affect the ability of supply chain members to bear blockchain variable costs. For example, since the manufacturer has certain price and channel advantages under the online direct selling mode, the manufacturer has a stronger ability to bear the variable cost of blockchain technology under the online direct selling mode.

Consumer channel preferences also affect the ability of supply chain members to bear the variable costs of blockchain technology. For example, when consumers prefer traditional channels, because traditional retailers have greater channel advantages, the increase in demand and retail price will increase profits. Therefore, when manufacturers adopt blockchain technology, traditional retailers are better able to share.

Response to Reviewer 2 Comments

Dear reviewer,

Thank you very much for your kind review and suggestions. According to your suggestions, we have made careful modification of our manuscript.

Thank you again for your positive comments and valuable suggestions to improve the quality of our manuscript.

With kind regards.

Di Wang

**Point 1:** The abstract should include a few preliminary sentences to highlight the importance of the topic. The authors start with the main concentration of the study immediately.

**Response 1:** Thank you for your positive comments and valuable suggestions to improve the quality of our manuscript. We have revised the abstract part of this paper based on the motivation and innovation points of this study.

**Point 2:** The introduction section could benefit from more statistical information to better highlight the importance of the main subject at hand.

**Response 2:** Thank you for your positive comments. We have added some statistics to the introduction of the article, the specific details of which are in the revised manuscript.

**Point 3:** The literature review: please double check for the most recent and relevant studies published over the last 2-3 years.

**Response 3:** Thank you for the comment. We have added the latest references to the revised manuscript.

**Point 4:** The model assumptions are presented well. I see that the adopted assumptions are supported by the relevant references. This will help justifying the adoption of these assumptions.

**Response 4:** Thank you for your nice comment.

**Point 5:** The presentation of the proposed solution methodology seems to be adequate. I suggest adding more supporting references for the key mathematical relationships used.

**Response 5:** Thank you for your comments and suggestions. In the revised version we have added more supporting references for the key mathematical relationships used.

**Point 6:** Please provide more details regarding the input data used throughout this study. More supporting references would be helpful to justify the data selection.

**Response 6:** Thank you for your comments and suggestions. We have revised the numerical analysis section of this article and added relevant references.

**Point 7:** The manuscript contains quite a lot of figures and tables. Please double check and try to provide a more detailed description of these figures and tables where appropriate to make sure that the future readers will have a reasonable understanding of what these figures and tables represent.

**Response 7:** Thank you for the comment. We have examined and revised the figures and tables that appear in the text, as can be found in the revised manuscript.

**Point 8:** The section devoted to numerical experiments should be expanded. It appears to be very short. The authors should include more analyses and discussions.

**Response 8:** Thank you for your comments and suggestions. We have added Figures 2-4 in the numerical analysis section of this paper to study the impact of relevant parameters on the profits of supply chain members under different models. Among them, figure 2-4 respectively when the other parameters must be explored, consumer preference for traditional channels, the level of blockchain technology investment, blockchain technology cost sensitivity influence on profit of supply chain members. expanded and discussed the numerical experiments section of this article.
