# Supplementary material for: Pricing decision and channel selection of fresh agricultural products dual-channel supply chain based on blockchain
Source: PLoS One. 2024 Mar 28;19(3):e0297484. doi: 10.1371/journal.pone.0297484 (PMC10977692; doi:10.1371/journal.pone.0297484)
Supplement: Attachment — Submitted filename: 20231114Comments.docx [file pone.0297484.s003.docx]

The manuscript shows several flaws.

1. There are several empty cells in table 1 and it doesn't look nice. Is it appropriate to fill in the left slash? Please adjust the width of each column of the table 1. For example, the first column should be narrower, while the second column may be wider.
2. The font of the word “section” on line 191 is inconsistent with others.
3. Fig 1 shows some flaws. The most fatal point is that the third and fourth subfigures fail to show any difference from the first and second subfigures. I suggest that the author include text boxes and arrows in the latter two subfigures to show the adoption of blockchain. Then, the four subfigures (a), (b), (c), (d) are not in Times New Roman. Last, $w_{r}^{BD}$ in the fourth subfigure is bolded, while others are not.
4. In line 253, the function includes exponential terms $e^{\frac{ln2}{T}t}$. For better readability, I suggest replacing it with $exp(\frac{ln2}{T}t)$.
5. The first column of table 3 shows NS*, while the third column is ND model. Similar problem exists in table 4.
6. Fig 5 shows weak resolvability. Please revise it to make the color difference more noticeable. Even it is obvious of Fig 6, please fill 3 different colors. Also, the size should be revised.
7. Some references are wrong.
1) Journal names are not all capitalized of 4, 13, 17, 23.
2) All words of the title are capitalized of 6, 38, 43, 59, 60.
3) Journal name has extra space of 8.
4) There is no uppercase after the colon of 9, 37.
5) The journal name is wrong of 12, 26, 30, and computer should be computers.
6) & is used in some references to link authors (for example 14), but others are not (for example 15).
7) The journal's name is abbreviated of 22.
8) Colon of the journal name is missing in 42.
