# Supplementary material for: Pricing decision and channel selection of fresh agricultural products dual-channel supply chain based on blockchain
Source: PLoS One. 2024 Mar 28;19(3):e0297484. doi: 10.1371/journal.pone.0297484 (PMC10977692; doi:10.1371/journal.pone.0297484)
Supplement: Attachment — Submitted filename: Review opinions.docx [file pone.0297484.s004.docx]

**Point 1:** The statement of the research problem in the introduction is not clear. Judging from the title, the research objective of this paper should be pricing strategy and channel selection, but the paper does not explain why it needs to conduct pricing strategy and channel selection, and the practical and theoretical significance of the research is not clear (Does the producer company in reality have a practical demand for price decision and channel responsibility based on blockchain technology’s dual-channel supply chain? Are there deficiencies and gaps in existing research in this area?). The innovative points and contributions of the research in the introduction are not clearly explained.

**Point 2:** The statement of the research problem in the introduction is not clear. Judging from the title, the research objective of this paper should be pricing strategy and channel selection, but the paper does not explain why it needs to conduct pricing strategy and channel selection, and the practical and theoretical significance of the research is not clear (Does the producer company in reality have a practical demand for price decision and channel responsibility based on blockchain technology’s dual-channel supply chain? Are there deficiencies and gaps in existing research in this area?). The innovative points and contributions of the research in the introduction are not clearly explained.

**Point 3:** The assumptions of the model in Chapter 3 are the same as the dual-channel model in reference [43]. It is recommended to clarify the differences between the existing study [43] and this research in the literature review section, which could highlight the innovation and contributions of this paper.

**Point 4:** What assumption from reference [27] is referenced in the numerical analysis section? What actual situation is it based on? Please provide a detailed explanation, as this will demonstrate the rationality of the numerical analysis.

**Point 5:** The conclusion of the article is almost consistent with the results of reference [43], offering no new findings, and fails to reflect the innovation and contribution of this study in the theoretical aspect.
