# Supplementary material for: Pricing decision and channel selection of fresh agricultural products dual-channel supply chain based on blockchain
Source: PLoS One. 2024 Mar 28;19(3):e0297484. doi: 10.1371/journal.pone.0297484 (PMC10977692; doi:10.1371/journal.pone.0297484)
Supplement: Attachment — Submitted filename: Response to Reviewers.docx [file pone.0297484.s005.docx]

Response to Reviewer #3 Comments

Dear reviewer,

Thank you very much for your kind review and suggestions. According to your suggestions, we have made careful modification of our manuscript.

Thank you again for your positive comments and valuable suggestions to improve the quality of our manuscript.

With kind regards.

Di Wang

1. There are several empty cells in table 1 and it doesn't look nice. Is it appropriate to fill in the left slash? Please adjust the width of each column of the table 1. For example, the first column should be narrower, while the second column may be wider.

**Response 1:** Thank you for your comments. We have filled every empty cells in table 1 with a left slash and adjusted the width of each column to make the table 1 look more comfortable.

2. The font of the word “section” on line 191 is inconsistent with others.

**Response 2:** Thank you very much for pointing out this problem, we have changed the font of the word “section” on line 191 to Times New Roman used in this article.

3. Fig 1 shows some flaws. The most fatal point is that the third and fourth subfigures fail to show any difference from the first and second subfigures. I suggest that the author include text boxes and arrows in the latter two subfigures to show the adoption of blockchain. Then, the four subfigures (a), (b), (c), (d) are not in Times New Roman. Last, $w_{r}^{BD}$ in the fourth subfigure is bolded, while others are not.

**Response 3:** Thanks for your comments, we have fixed the defect in Fig 1. First, we added blockchain technology section at the top of the latter two subfigures in the form of text boxes and arrows to show the adoption of blockchain. Second, we change the four subfigures (a), (b), (c), and (d) to Times New Roman. Finally, the bold form of $w_{r}^{BD}$ in the fourth subfigure is also removed to keep it consistent with others.

4. In line 253, the function includes exponential terms $e^{\frac{ln2}{T}t}$. For better readability, I suggest replacing it with $exp(\frac{ln2}{T}t)$.

**Response 4:** Thank you very much for your comments, and we have adopted your suggestion to replace the function includes exponential terms $e^{\frac{ln2}{T}t}$ in line 253 with $exp(\frac{ln2}{T}t)$.

5. The first column of table 3 shows NS*, while the third column is ND model. Similar problem exists in table 4.

**Response 5:** Thank you very much for your comments. We have modified the symbols in the first column of table 3 and table 4 accordingly.

6. Fig 5 shows weak resolvability. Please revise it to make the color difference more noticeable. Even it is obvious of Fig 6, please fill 3 different colors. Also, the size should be revised.

**Response 6:** Thank you for your suggestions. We have modified Fig 5 and Fig 6 according to your suggestions in the revised manuscript.

7. Some references are wrong.
1) Journal names are not all capitalized of 4, 13, 17, 23.
2) All words of the title are capitalized of 6, 38, 43, 59, 60.
3) Journal name has extra space of 8.
4) There is no uppercase after the colon of 9, 37.
5) The journal name is wrong of 12, 26, 30, and computer should be computers.
6) & is used in some references to link authors (for example 14), but others are not (for example 15).
7) The journal's name is abbreviated of 22.
8) Colon of the journal name is missing in 42.

**Response 7:** Thank you very much for your suggestions. We have revised the problems in the references you raised one by one.

Response to Reviewer #4 Comments

Dear reviewer,

Thank you very much for your kind review and suggestions. According to your suggestions, we have made careful modification of our manuscript.

Thank you again for your positive comments and valuable suggestions to improve the quality of our manuscript.

With kind regards.

Di Wang

**Point 1:** The statement of the research problem in the introduction is not clear. Judging from the title, the research objective of this paper should be pricing strategy and channel selection, but the paper does not explain why it needs to conduct pricing strategy and channel selection, and the practical and theoretical significance of the research is not clear (Does the producer company in reality have a practical demand for price decision and channel responsibility based on blockchain technology’s dual-channel supply chain? Are there deficiencies and gaps in existing research in this area?). The innovative points and contributions of the research in the introduction are not clearly explained.

**Response 1:** Thank you for your comments. According to your suggestions, we have made modifications to the introduction and literature review sections of this paper. In the introduction, we explain the reasons for conducting research on pricing strategies and channel selection, and have included company examples to clarify the significance, innovation, and contribution of this study. In the literature review section, we provide an overview of research on pricing decisions and channel selection in the dual-channel supply chain of fresh agricultural products, summarizing the differences between this paper and existing literature. The specific reasons, significance, and innovative aspects of this paper's study on the pricing strategies and channel selection of the dual-channel supply chain for fresh agricultural products under blockchain technology are as follows.

With the development of the online retail market, the integration of online and offline is becoming increasingly mature. Fresh agricultural product manufacturers, especially those in agricultural industrialization and professional cooperatives, to expand their presence beyond traditional retail channels by venturing into the online sphere, aiming to enhance the quality of agricultural products and capture a larger market share. High-quality agricultural product manufacturers such as Anchor, Dole, and Jiawo adopt the online direct sales model, while some smaller-scale agricultural product manufacturers choose the online distribution model. The sales model based on a dual-channel structure will reduce the sales costs for enterprises and increase their market share, thereby benefiting the business. However, the diversification of dual-channel structures, intense channel competition, easily trigger "free-riding" behavior and vicious price competition. In addition, consumer channel preferences can also have a significant impact on the management decisions of supply chain enterprises, such as influencing the price competition relationships among businesses. In this sense, it is crucial to study the pricing and channel selection issues of the dual-channel supply chain of fresh agricultural products composed of manufacturers and retailers from a systemic perspective.

Additionally, through the analysis of relevant literature, we have found that existing studies often approach blockchain technology and the fresh agricultural product supply chain from a singular perspective, with limited literature quantifying the impact of blockchain technology. Therefore, this paper focuses on a second-tier fresh agricultural product supply chain composed of individual suppliers and retailers. It considers factors such as the circulation efficiency of fresh agricultural products, blockchain unit variable costs, the level of investment in blockchain technology, and consumer channel preferences. The paper employs methods such as model construction and numerical analysis to study the pricing and channel selection issues in a dual-channel supply chain for fresh agricultural products based on blockchain technology. The research conclusions can provide management and decision-making recommendations for different supply chain participants and offer insights and methods for addressing the aforementioned issues.

In the literature review section, we conducted a comparative analysis of the achievements in pricing decisions and channel selection in the dual-channel supply chain for fresh agricultural products. However, there are still gaps in the existing literature in the following aspects. Firstly, existing research has focused on pricing and coordination issues in single-channel or single dual-channel mode supply chains for fresh agricultural products, and there is a need for additional research on pricing and channel selection in dual-channel supply chains for fresh agricultural products under different dual-channel structural models. Secondly, most scholars have concentrated on case studies to explore the impact of blockchain on business operations and decision-making. There is a lack of models quantifying the economic benefits of blockchain technology in improving the circulation time of fresh agricultural products and increasing consumer trust under blockchain models. Thirdly, existing literature primarily studies the impact of blockchain technology on the supply chain from a singular perspective, with relatively few results considering the combined influence of consumer preferences, the level of blockchain utilization, and blockchain variable costs on simulation outcomes.

**Point 2:** The statement of the research problem in the introduction is not clear. Judging from the title, the research objective of this paper should be pricing strategy and channel selection, but the paper does not explain why it needs to conduct pricing strategy and channel selection, and the practical and theoretical significance of the research is not clear (Does the producer company in reality have a practical demand for price decision and channel responsibility based on blockchain technology’s dual-channel supply chain? Are there deficiencies and gaps in existing research in this area?). The innovative points and contributions of the research in the introduction are not clearly explained.

**Response 2:** Thank you for your comments. According to your suggestions, we have made modifications to the introduction and literature review sections of this paper. In the introduction, we explain the reasons for conducting research on pricing strategies and channel selection, and have included company examples to clarify the significance, innovation, and contribution of this study. In the literature review section, we provide an overview of research on pricing decisions and channel selection in the dual-channel supply chain of fresh agricultural products, summarizing the differences between this paper and existing literature. The specific reasons, significance, and innovative aspects of this paper's study on the pricing strategies and channel selection of the dual-channel supply chain for fresh agricultural products under blockchain technology are as follows.

With the development of the online retail market, the integration of online and offline is becoming increasingly mature. Fresh agricultural product manufacturers, especially those in agricultural industrialization and professional cooperatives, to expand their presence beyond traditional retail channels by venturing into the online sphere, aiming to enhance the quality of agricultural products and capture a larger market share. High-quality agricultural product manufacturers such as Anchor, Dole, and Jiawo adopt the online direct sales model, while some smaller-scale agricultural product manufacturers choose the online distribution model. The sales model based on a dual-channel structure will reduce the sales costs for enterprises and increase their market share, thereby benefiting the business. However, the diversification of dual-channel structures, intense channel competition, easily trigger "free-riding" behavior and vicious price competition. In addition, consumer channel preferences can also have a significant impact on the management decisions of supply chain enterprises, such as influencing the price competition relationships among businesses. In this sense, it is crucial to study the pricing and channel selection issues of the dual-channel supply chain of fresh agricultural products composed of manufacturers and retailers from a systemic perspective.

Additionally, through the analysis of relevant literature, we have found that existing studies often approach blockchain technology and the fresh agricultural product supply chain from a singular perspective, with limited literature quantifying the impact of blockchain technology. Therefore, this paper focuses on a second-tier fresh agricultural product supply chain composed of individual suppliers and retailers. It considers factors such as the circulation efficiency of fresh agricultural products, blockchain unit variable costs, the level of investment in blockchain technology, and consumer channel preferences. The paper employs methods such as model construction and numerical analysis to study the pricing and channel selection issues in a dual-channel supply chain for fresh agricultural products based on blockchain technology. The research conclusions can provide management and decision-making recommendations for different supply chain participants and offer insights and methods for addressing the aforementioned issues.

In the literature review section, we conducted a comparative analysis of the achievements in pricing decisions and channel selection in the dual-channel supply chain for fresh agricultural products. However, there are still gaps in the existing literature in the following aspects. Firstly, existing research has focused on pricing and coordination issues in single-channel or single dual-channel mode supply chains for fresh agricultural products, and there is a need for additional research on pricing and channel selection in dual-channel supply chains for fresh agricultural products under different dual-channel structural models. Secondly, most scholars have concentrated on case studies to explore the impact of blockchain on business operations and decision-making. There is a lack of models quantifying the economic benefits of blockchain technology in improving the circulation time of fresh agricultural products and increasing consumer trust under blockchain models. Thirdly, existing literature primarily studies the impact of blockchain technology on the supply chain from a singular perspective, with relatively few results considering the combined influence of consumer preferences, the level of blockchain utilization, and blockchain variable costs on simulation outcomes.

**Point 3:** The assumptions of the model in Chapter 3 are the same as the dual-channel model in reference [43]. It is recommended to clarify the differences between the existing study [43] and this research in the literature review section, which could highlight the innovation and contributions of this paper.

**Response 3:** Thank you for your suggestions, and we have clarified the differences between this article and reference [43] in the literature review section of the revised manuscript. This is specifically reflected in the following three aspects:

(1) The research focus of this article differs from that of reference [43]. Our study primarily targets the supply chain of fresh agricultural products, introducing relevant parameters closely associated with the characteristics of fresh agricultural products, such as circulation time, freshness, and the ratio of effective output.

(2) Unlike reference [43], this article comprehensively considers factors such as cross-price elasticity, consumer channel preferences, and online direct selling costs, all of which influence decision-making in dual-channel supply chains.

(3) While reference [43] only considers the fixed costs associated with the introduction of blockchain technology, this article introduces the degree of blockchain usage. Furthermore, we incorporate blockchain technology's variable costs and consumer trust gains, emphasizing their significant impact on the adoption decision of blockchain technology.

**Point 4:** What assumption from reference [27] is referenced in the numerical analysis section? What actual situation is it based on? Please provide a detailed explanation, as this will demonstrate the rationality of the numerical analysis.

**Response 4:** Thank you for your comments. Special note: Due to revisions made to the article, reference [27] has been updated to reference [34]. This paper mainly cites the data of circulation time, production and transportation cost of fresh agricultural products in reference [34], and these data are based on the actual data and materials of a cherry manufacturer in Yantai, Shandong, China. We have made a detailed supplementary explanation of this part in the revised manuscript.

**Point 5:** The conclusion of the article is almost consistent with the results of reference [43], offering no new findings, and fails to reflect the innovation and contribution of this study in the theoretical aspect.

**Response 5:** Thank you for your comments. Based on the questions you raised, we have further summarized and supplemented the conclusion section of this paper. Additionally, we have presented some managerial insights derived from the research conclusions, enhancing the richness of the conclusion section.

Response to Reviewer #6 Comments

Dear reviewer,

Thank you very much for your kind review and suggestions. According to your suggestions, we have made careful modification of our manuscript.

Thank you again for your positive comments and valuable suggestions to improve the quality of our manuscript.

With kind regards.

Di Wang

1. The impact of information asymmetry in the supply chain on fresh produce is not clearly described in the introduction.

**Response 1:** Thanks for your comments. According to your suggestion, we have made a supplementary explanation on the impact of information asymmetry in the supply chain on fresh agricultural products in the introduction of this paper. With the circulation of products, product quality information will continue to decay, resulting in serious information asymmetry in the process of product quality supervision. Consumers in the inferior position of information can not trace the product quality, and the rights and interests of consumers can not be protected. In the traditional mode, the information of product quality traceability comes from the manufacturer or the third party enterprise. They may arbitrarily change the product information in order to pursue the maximization of benefits, which leads to the failure of consumers to effectively identify the product quality, and thus leads to the "trust crisis" of consumers on the disclosure of information by enterprises.

2. In the literature review section, it is recommended to add a table that compares the existing research with the research focus of this article to highlight the novelty of the article.

**Response 2:** Thank you for your suggestions. We have conducted a comparative analysis of relevant literature, and the results are presented in Table 1. Through this comparative analysis, we have identified several shortcomings in existing literature:

Firstly, previous research has mainly focused on pricing and coordination issues in the supply chain of fresh agricultural products under single-channel or single dual-channel models. There is a need for additional studies addressing the pricing and channel selection problems in the dual-channel supply chain of fresh agricultural products under different dual-channel structural models.

Secondly, most scholars have concentrated on using case studies to explore the impact of blockchain on enterprise operations and decision-making. However, there is a lack of models quantifying the economic benefits of blockchain technology in improving the circulation time of fresh agricultural products and increasing consumer trust under a blockchain paradigm.

Thirdly, existing literature primarily examines the impact of blockchain technology on the supply chain from a singular perspective. There is relatively less research that comprehensively considers the impact of consumer preferences, the degree of blockchain usage, and the variable costs of blockchain on simulation results.

In this paper, we focus on a dual-channel supply chain of fresh agricultural products consisting of one supplier and one retailer. We introduce parameters such as the circulation efficiency of fresh agricultural products, variable costs of blockchain units, the level of investment in blockchain technology, and consumer channel preferences. Using the Stackelberg game model, we conduct a comparative analysis of the pricing and channel selection strategies in the dual-channel supply chain of fresh agricultural products under two scenarios: without using blockchain technology and adopting blockchain technology.

3. The model description is not clear, and the circulation time of the product should be t0 , when blockchain technology is not used. And please explain why the circulation time of the product changes after using blockchain technology.

**Response 3:** Thanks for your comments and we have made corresponding modifications in Assumption 1. In this assumption, the circulation time of the products is assumed to be $t_{0}$, when blockchain technology is not used. After the adoption of blockchain technology, mainly through the traceability system of blockchain, automatic identification of items and automatic collection of data can be successfully completed. Therefore, it can speed up the speed of logistics links, reduce invalid paths, and shorten the circulation time of products in upstream and downstream enterprises. Accordingly, the circulation time will change from the original $t_{0}$ to $t_{1}$, $t_{0}>t_{1}$, effectively improve the freshness of the product and effective output ratio.

4. Whether the parameter settings in the numerical study have a corresponding practical basis or theoretical support.

**Response 4:** Thank you for your comments. We have added a corresponding practical basis for parameter setting in numerical research in the revised manuscript.

5. The conclusion of the article is less content, and it is recommended to enrich the relevant content.↳

**Response 5:** Thanks for your comments, we have made corresponding contents to the conclusion of this article in the revised manuscript.
